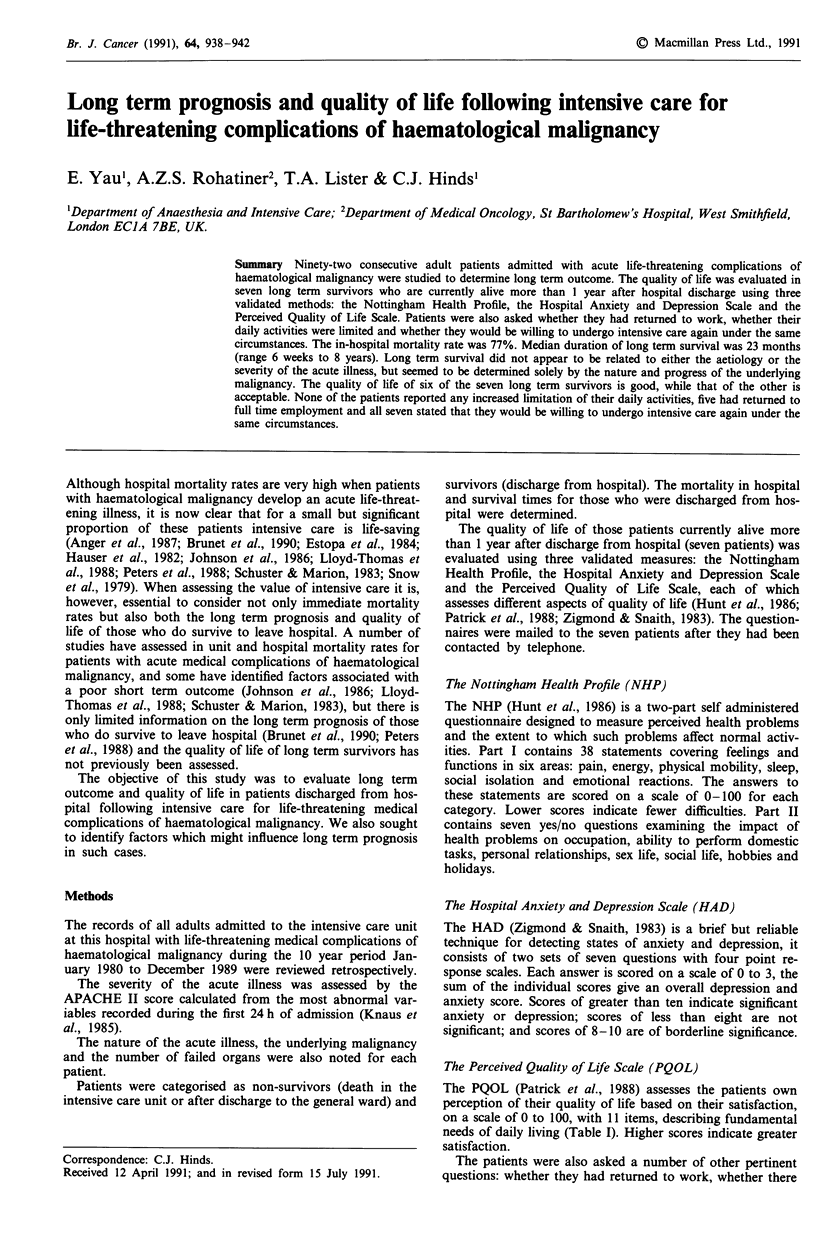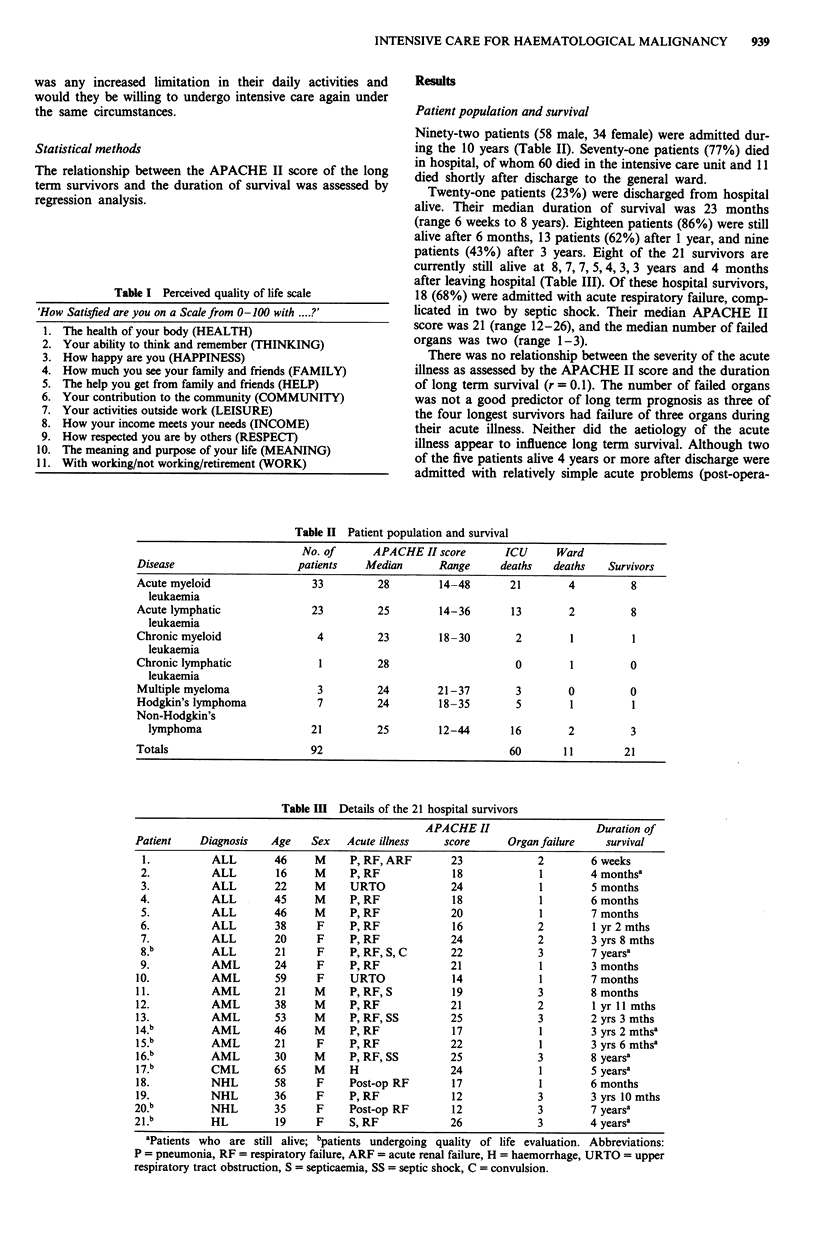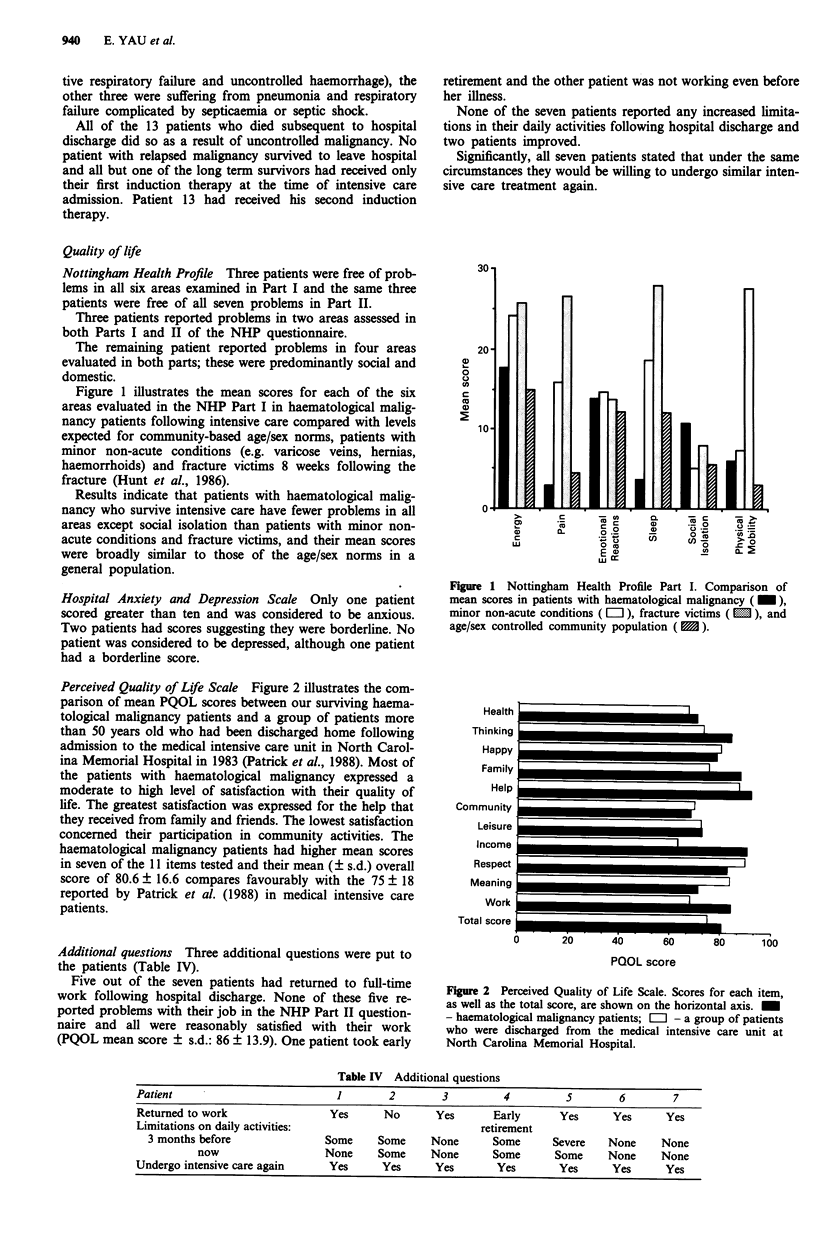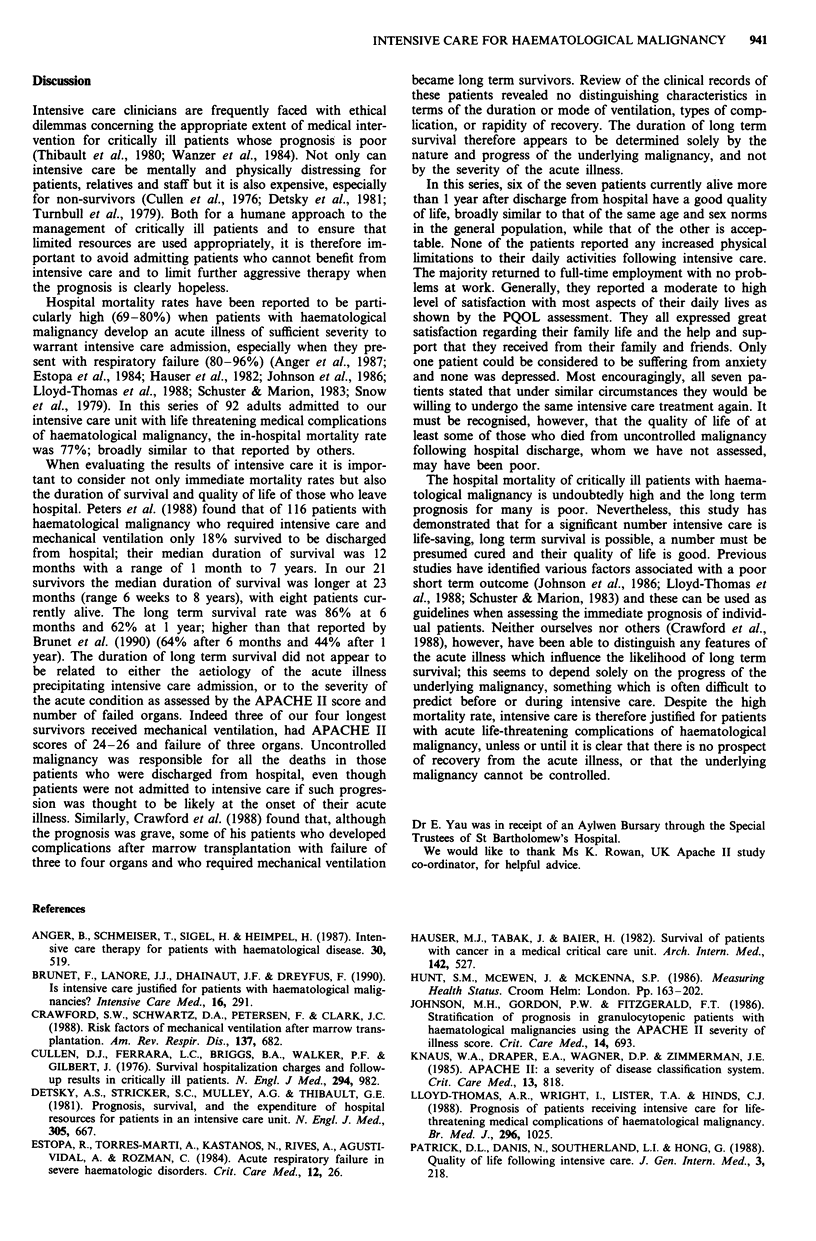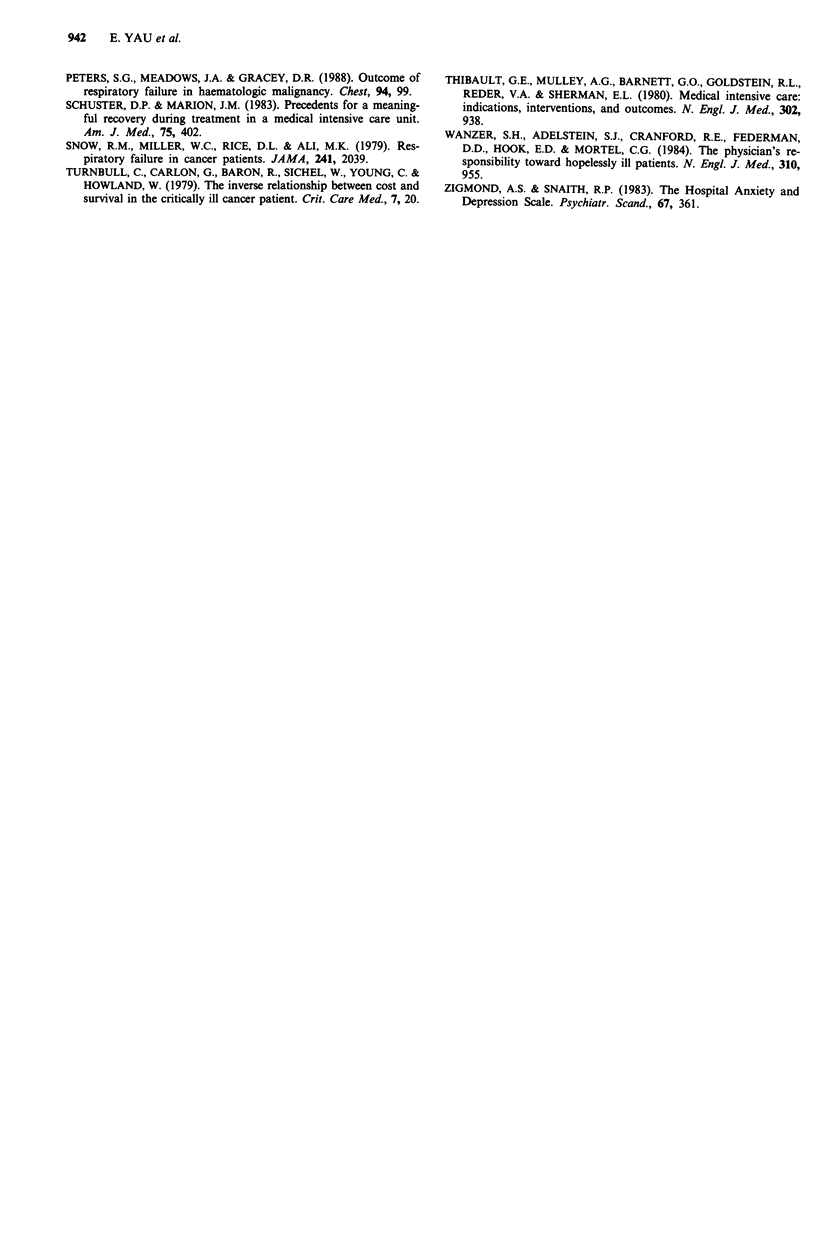# Long term prognosis and quality of life following intensive care for life-threatening complications of haematological malignancy.

**DOI:** 10.1038/bjc.1991.430

**Published:** 1991-11

**Authors:** E. Yau, A. Z. Rohatiner, T. A. Lister, C. J. Hinds

**Affiliations:** Department of Anaesthesia and Intensive Care, St Bartholomew's Hospital, West Smithfield, London, UK.

## Abstract

Ninety-two consecutive adult patients admitted with acute life-threatening complications of haematological malignancy were studied to determine long term outcome. The quality of life was evaluated in seven long term survivors who are currently alive more than 1 year after hospital discharge using three validated methods: the Nottingham Health Profile, the Hospital Anxiety and Depression Scale and the Perceived Quality of Life Scale. Patients were also asked whether they had returned to work, whether their daily activities were limited and whether they would be willing to undergo intensive care again under the same circumstances. The in-hospital mortality rate was 77%. Median duration of long term survival was 23 months (range 6 weeks to 8 years). Long term survival did not appear to be related to either the aetiology or the severity of the acute illness, but seemed to be determined solely by the nature and progress of the underlying malignancy. The quality of life of six of the seven long term survivors is good, while that of the other is acceptable. None of the patients reported any increased limitation of their daily activities, five had returned to full time employment and all seven stated that they would be willing to undergo intensive care again under the same circumstances.


Br. J. Cancer (1991), 64, 938-942 © Macmillan Press Ltd., 1991

### Long term prognosis and quality of life following intensive care for life-threatening complications of haematological malignancy

E. Yau[1], A.Z.S. Rohatiner[2], T.A. Lister & C.J. Hinds[1]

[1]*Department of Anaesthesia and Intensive Care;* [2]*Department of Medical Oncology, St Bartholomew's Hospital, West Smithfield, London EC1A 7BE, UK.*

**Summary** Ninety-two consecutive adult patients admitted with acute life-threatening complications of haematological malignancy were studied to determine long term outcome. The quality of life was evaluated in seven long term survivors who are currently alive more than 1 year after hospital discharge using three validated methods: the Nottingham Health Profile, the Hospital Anxiety and Depression Scale and the Perceived Quality of Life Scale. Patients were also asked whether they had returned to work, whether their daily activities were limited and whether they would be willing to undergo intensive care again under the same circumstances. The in-hospital mortality rate was 77%. Median duration of long term survival was 23 months (range 6 weeks to 8 years). Long term survival did not appear to be related to either the aetiology or the severity of the acute illness, but seemed to be determined solely by the nature and progress of the underlying malignancy. The quality of life of six of the seven long term survivors is good, while that of the other is acceptable. None of the patients reported any increased limitation of their daily activities, five had returned to full time employment and all seven stated that they would be willing to undergo intensive care again under the same circumstances.

Although hospital mortality rates are very high when patients with haematological malignancy develop an acute life-threatening illness, it is now clear that for a small but significant proportion of these patients intensive care is life-saving (Anger *et al.*, 1987; Brunet *et al.*, 1990; Estopa *et al.*, 1984; Hauser *et al.*, 1982; Johnson *et al.*, 1986; Lloyd-Thomas *et al.*, 1988; Peters *et al.*, 1988; Schuster & Marion, 1983; Snow *et al.*, 1979). When assessing the value of intensive care it is, however, essential to consider not only immediate mortality rates but also both the long term prognosis and quality of life of those who do survive to leave hospital. A number of studies have assessed in unit and hospital mortality rates for patients with acute medical complications of haematological malignancy, and some have identified factors associated with a poor short term outcome (Johnson *et al.*, 1986; Lloyd-Thomas *et al.*, 1988; Schuster & Marion, 1983), but there is only limited information on the long term prognosis of those who do survive to leave hospital (Brunet *et al.*, 1990; Peters *et al.*, 1988) and the quality of life of long term survivors has not previously been assessed.

The objective of this study was to evaluate long term outcome and quality of life in patients discharged from hospital following intensive care for life-threatening medical complications of haematological malignancy. We also sought to identify factors which might influence long term prognosis in such cases.

### Methods

The records of all adults admitted to the intensive care unit at this hospital with life-threatening medical complications of haematological malignancy during the 10 year period January 1980 to December 1989 were reviewed retrospectively.

The severity of the acute illness was assessed by the APACHE II score calculated from the most abnormal variables recorded during the first 24 h of admission (Knaus *et al.*, 1985).

The nature of the acute illness, the underlying malignancy and the number of failed organs were also noted for each patient.

Patients were categorised as non-survivors (death in the intensive care unit or after discharge to the general ward) and survivors (discharge from hospital). The mortality in hospital and survival times for those who were discharged from hospital were determined.

The quality of life of those patients currently alive more than 1 year after discharge from hospital (seven patients) was evaluated using three validated measures: the Nottingham Health Profile, the Hospital Anxiety and Depression Scale and the Perceived Quality of Life Scale, each of which assesses different aspects of quality of life (Hunt *et al.*, 1986; Patrick *et al.*, 1988; Zigmond & Snaith, 1983). The questionnaires were mailed to the seven patients after they had been contacted by telephone.

### *The Nottingham Health Profile (NHP)*

The NHP (Hunt *et al.*, 1986) is a two-part self administered questionnaire designed to measure perceived health problems and the extent to which such problems affect normal activities. Part I contains 38 statements covering feelings and functions in six areas: pain, energy, physical mobility, sleep, social isolation and emotional reactions. The answers to these statements are scored on a scale of 0-100 for each category. Lower scores indicate fewer difficulties. Part II contains seven yes/no questions examining the impact of health problems on occupation, ability to perform domestic tasks, personal relationships, sex life, social life, hobbies and holidays.

### *The Hospital Anxiety and Depression Scale (HAD)*

The HAD (Zigmond & Snaith, 1983) is a brief but reliable technique for detecting states of anxiety and depression, it consists of two sets of seven questions with four point response scales. Each answer is scored on a scale of 0 to 3, the sum of the individual scores give an overall depression and anxiety score. Scores of greater than ten indicate significant anxiety or depression; scores of less than eight are not significant; and scores of 8-10 are of borderline significance.

### *The Perceived Quality of Life Scale (PQOL)*

The PQOL (Patrick *et al.*, 1988) assesses the patients own perception of their quality of life based on their satisfaction, on a scale of 0 to 100, with 11 items, describing fundamental needs of daily living (Table I). Higher scores indicate greater satisfaction.

The patients were also asked a number of other pertinent questions: whether they had returned to work, whether there

Correspondence: C.J. Hinds.
Received 12 April 1991; and in revised form 15 July 1991.

was any increased limitation in their daily activities and would they be willing to undergo intensive care again under the same circumstances.

*Statistical methods*

The relationship between the APACHE II score of the long term survivors and the duration of survival was assessed by regression analysis.

**Table I** Perceived quality of life scale

| *'How Satisfied are you on a Scale from 0–100 with ....?'* |
|---|
| 1. The health of your body (HEALTH) |
| 2. Your ability to think and remember (THINKING) |
| 3. How happy are you (HAPPINESS) |
| 4. How much you see your family and friends (FAMILY) |
| 5. The help you get from family and friends (HELP) |
| 6. Your contribution to the community (COMMUNITY) |
| 7. Your activities outside work (LEISURE) |
| 8. How your income meets your needs (INCOME) |
| 9. How respected you are by others (RESPECT) |
| 10. The meaning and purpose of your life (MEANING) |
| 11. With working/not working/retirement (WORK) |

### Results

*Patient population and survival*

Ninety-two patients (58 male, 34 female) were admitted during the 10 years (Table II). Seventy-one patients (77%) died in hospital, of whom 60 died in the intensive care unit and 11 died shortly after discharge to the general ward.

Twenty-one patients (23%) were discharged from hospital alive. Their median duration of survival was 23 months (range 6 weeks to 8 years). Eighteen patients (86%) were still alive after 6 months, 13 patients (62%) after 1 year, and nine patients (43%) after 3 years. Eight of the 21 survivors are currently still alive at 8, 7, 7, 5, 4, 3, 3 years and 4 months after leaving hospital (Table III). Of these hospital survivors, 18 (68%) were admitted with acute respiratory failure, complicated in two by septic shock. Their median APACHE II score was 21 (range 12–26), and the median number of failed organs was two (range 1–3).

There was no relationship between the severity of the acute illness as assessed by the APACHE II score and the duration of long term survival ($r = 0.1$). The number of failed organs was not a good predictor of long term prognosis as three of the four longest survivors had failure of three organs during their acute illness. Neither did the aetiology of the acute illness appear to influence long term survival. Although two of the five patients alive 4 years or more after discharge were admitted with relatively simple acute problems (post-opera-

**Table II** Patient population and survival

| *Disease* | *No. of patients* | *APACHE II score Median* | *Range* | *ICU deaths* | *Ward deaths* | *Survivors* |
|---|---|---|---|---|---|---|
| Acute myeloid leukaemia | 33 | 28 | 14–48 | 21 | 4 | 8 |
| Acute lymphatic leukaemia | 23 | 25 | 14–36 | 13 | 2 | 8 |
| Chronic myeloid leukaemia | 4 | 23 | 18–30 | 2 | 1 | 1 |
| Chronic lymphatic leukaemia | 1 | 28 | | 0 | 1 | 0 |
| Multiple myeloma | 3 | 24 | 21–37 | 3 | 0 | 0 |
| Hodgkin's lymphoma | 7 | 24 | 18–35 | 5 | 1 | 1 |
| Non-Hodgkin's lymphoma | 21 | 25 | 12–44 | 16 | 2 | 3 |
| Totals | 92 | | | 60 | 11 | 21 |

**Table III** Details of the 21 hospital survivors

| *Patient* | *Diagnosis* | *Age* | *Sex* | *Acute illness* | *APACHE II score* | *Organ failure* | *Duration of survival* |
|---|---|---|---|---|---|---|---|
| 1. | ALL | 46 | M | P, RF, ARF | 23 | 2 | 6 weeks |
| 2. | ALL | 16 | M | P, RF | 18 | 1 | 4 months[a] |
| 3. | ALL | 22 | M | URTO | 24 | 1 | 5 months |
| 4. | ALL | 45 | M | P, RF | 18 | 1 | 6 months |
| 5. | ALL | 46 | M | P, RF | 20 | 1 | 7 months |
| 6. | ALL | 38 | F | P, RF | 16 | 2 | 1 yr 2 mths |
| 7. | ALL | 20 | F | P, RF | 24 | 2 | 3 yrs 8 mths |
| 8.[b] | ALL | 21 | F | P, RF, S, C | 22 | 3 | 7 years[a] |
| 9. | AML | 24 | F | P, RF | 21 | 1 | 3 months |
| 10. | AML | 59 | F | URTO | 14 | 1 | 7 months |
| 11. | AML | 21 | M | P, RF, S | 19 | 3 | 8 months |
| 12. | AML | 38 | M | P, RF | 21 | 2 | 1 yr 11 mths |
| 13. | AML | 53 | M | P, RF, SS | 25 | 3 | 2 yrs 3 mths |
| 14.[b] | AML | 46 | M | P, RF | 17 | 1 | 3 yrs 2 mths[a] |
| 15.[b] | AML | 21 | F | P, RF | 22 | 1 | 3 yrs 6 mths[a] |
| 16.[b] | AML | 30 | M | P, RF, SS | 25 | 3 | 8 years[a] |
| 17.[b] | CML | 65 | M | H | 24 | 1 | 5 years[a] |
| 18. | NHL | 58 | F | Post-op RF | 17 | 1 | 6 months |
| 19. | NHL | 36 | F | P, RF | 12 | 3 | 3 yrs 10 mths |
| 20.[b] | NHL | 35 | F | Post-op RF | 12 | 3 | 7 years[a] |
| 21.[b] | HL | 19 | F | S, RF | 26 | 3 | 4 years[a] |

[a]Patients who are still alive; [b]patients undergoing quality of life evaluation. Abbreviations: P = pneumonia, RF = respiratory failure, ARF = acute renal failure, H = haemorrhage, URTO = upper respiratory tract obstruction, S = septicaemia, SS = septic shock, C = convulsion.

tive respiratory failure and uncontrolled haemorrhage), the other three were suffering from pneumonia and respiratory failure complicated by septicaemia or septic shock.

All of the 13 patients who died subsequent to hospital discharge did so as a result of uncontrolled malignancy. No patient with relapsed malignancy survived to leave hospital and all but one of the long term survivors had received only their first induction therapy at the time of intensive care admission. Patient 13 had received his second induction therapy.

*Quality of life*

*Nottingham Health Profile* Three patients were free of problems in all six areas examined in Part I and the same three patients were free of all seven problems in Part II.

Three patients reported problems in two areas assessed in both Parts I and II of the NHP questionnaire.

The remaining patient reported problems in four areas evaluated in both parts; these were predominantly social and domestic.

Figure 1 illustrates the mean scores for each of the six areas evaluated in the NHP Part I in haematological malignancy patients following intensive care compared with levels expected for community-based age/sex norms, patients with minor non-acute conditions (e.g. varicose veins, hernias, haemorrhoids) and fracture victims 8 weeks following the fracture (Hunt *et al.*, 1986).

Results indicate that patients with haematological malignancy who survive intensive care have fewer problems in all areas except social isolation than patients with minor non-acute conditions and fracture victims, and their mean scores were broadly similar to those of the age/sex norms in a general population.

*Hospital Anxiety and Depression Scale* Only one patient scored greater than ten and was considered to be anxious. Two patients had scores suggesting they were borderline. No patient was considered to be depressed, although one patient had a borderline score.

*Perceived Quality of Life Scale* Figure 2 illustrates the comparison of mean PQOL scores between our surviving haematological malignancy patients and a group of patients more than 50 years old who had been discharged home following admission to the medical intensive care unit in North Carolina Memorial Hospital in 1983 (Patrick *et al.*, 1988). Most of the patients with haematological malignancy expressed a moderate to high level of satisfaction with their quality of life. The greatest satisfaction was expressed for the help that they received from family and friends. The lowest satisfaction concerned their participation in community activities. The haematological malignancy patients had higher mean scores in seven of the 11 items tested and their mean (± s.d.) overall score of 80.6 ± 16.6 compares favourably with the 75 ± 18 reported by Patrick *et al.* (1988) in medical intensive care patients.

*Additional questions* Three additional questions were put to the patients (Table IV).

Five out of the seven patients had returned to full-time work following hospital discharge. None of these five reported problems with their job in the NHP Part II questionnaire and all were reasonably satisfied with their work (PQOL mean score ± s.d.: 86 ± 13.9). One patient took early retirement and the other patient was not working even before her illness.

None of the seven patients reported any increased limitations in their daily activities following hospital discharge and two patients improved.

Significantly, all seven patients stated that under the same circumstances they would be willing to undergo similar intensive care treatment again.

**Figure 1** Nottingham Health Profile Part I. Comparison of mean scores in patients with haematological malignancy ( ■ ), minor non-acute conditions ( □ ), fracture victims ( ▒ ), and age/sex controlled community population ( ▨ ).

**Figure 2** Perceived Quality of Life Scale. Scores for each item, as well as the total score, are shown on the horizontal axis. ■ – haematological malignancy patients; □ – a group of patients who were discharged from the medical intensive care unit at North Carolina Memorial Hospital.

**Table IV** Additional questions

| *Patient* | *1* | *2* | *3* | *4* | *5* | *6* | *7* |
|---|---|---|---|---|---|---|---|
| Returned to work | Yes | No | Yes | Early retirement | Yes | Yes | Yes |
| Limitations on daily activities: | | | | | | | |
| 3 months before | Some | Some | None | Some | Severe | None | None |
| now | None | Some | None | Some | Some | None | None |
| Undergo intensive care again | Yes | Yes | Yes | Yes | Yes | Yes | Yes |

### Discussion

Intensive care clinicians are frequently faced with ethical dilemmas concerning the appropriate extent of medical intervention for critically ill patients whose prognosis is poor (Thibault *et al.*, 1980; Wanzer *et al.*, 1984). Not only can intensive care be mentally and physically distressing for patients, relatives and staff but it is also expensive, especially for non-survivors (Cullen *et al.*, 1976; Detsky *et al.*, 1981; Turnbull *et al.*, 1979). Both for a humane approach to the management of critically ill patients and to ensure that limited resources are used appropriately, it is therefore important to avoid admitting patients who cannot benefit from intensive care and to limit further aggressive therapy when the prognosis is clearly hopeless.

Hospital mortality rates have been reported to be particularly high (69–80%) when patients with haematological malignancy develop an acute illness of sufficient severity to warrant intensive care admission, especially when they present with respiratory failure (80–96%) (Anger *et al.*, 1987; Estopa *et al.*, 1984; Hauser *et al.*, 1982; Johnson *et al.*, 1986; Lloyd-Thomas *et al.*, 1988; Schuster & Marion, 1983; Snow *et al.*, 1979). In this series of 92 adults admitted to our intensive care unit with life threatening medical complications of haematological malignancy, the in-hospital mortality rate was 77%; broadly similar to that reported by others.

When evaluating the results of intensive care it is important to consider not only immediate mortality rates but also the duration of survival and quality of life of those who leave hospital. Peters *et al.* (1988) found that of 116 patients with haematological malignancy who required intensive care and mechanical ventilation only 18% survived to be discharged from hospital; their median duration of survival was 12 months with a range of 1 month to 7 years. In our 21 survivors the median duration of survival was longer at 23 months (range 6 weeks to 8 years), with eight patients currently alive. The long term survival rate was 86% at 6 months and 62% at 1 year; higher than that reported by Brunet *et al.* (1990) (64% after 6 months and 44% after 1 year). The duration of long term survival did not appear to be related to either the aetiology of the acute illness precipitating intensive care admission, or to the severity of the acute condition as assessed by the APACHE II score and number of failed organs. Indeed three of our four longest survivors received mechanical ventilation, had APACHE II scores of 24–26 and failure of three organs. Uncontrolled malignancy was responsible for all the deaths in those patients who were discharged from hospital, even though patients were not admitted to intensive care if such progression was thought to be likely at the onset of their acute illness. Similarly, Crawford *et al.* (1988) found that, although the prognosis was grave, some of his patients who developed complications after marrow transplantation with failure of three to four organs and who required mechanical ventilation became long term survivors. Review of the clinical records of these patients revealed no distinguishing characteristics in terms of the duration or mode of ventilation, types of complication, or rapidity of recovery. The duration of long term survival therefore appears to be determined solely by the nature and progress of the underlying malignancy, and not by the severity of the acute illness.

In this series, six of the seven patients currently alive more than 1 year after discharge from hospital have a good quality of life, broadly similar to that of the same age and sex norms in the general population, while that of the other is acceptable. None of the patients reported any increased physical limitations to their daily activities following intensive care. The majority returned to full-time employment with no problems at work. Generally, they reported a moderate to high level of satisfaction with most aspects of their daily lives as shown by the PQOL assessment. They all expressed great satisfaction regarding their family life and the help and support that they received from their family and friends. Only one patient could be considered to be suffering from anxiety and none was depressed. Most encouragingly, all seven patients stated that under similar circumstances they would be willing to undergo the same intensive care treatment again. It must be recognised, however, that the quality of life of at least some of those who died from uncontrolled malignancy following hospital discharge, whom we have not assessed, may have been poor.

The hospital mortality of critically ill patients with haematological malignancy is undoubtedly high and the long term prognosis for many is poor. Nevertheless, this study has demonstrated that for a significant number intensive care is life-saving, long term survival is possible, a number must be presumed cured and their quality of life is good. Previous studies have identified various factors associated with a poor short term outcome (Johnson *et al.*, 1986; Lloyd-Thomas *et al.*, 1988; Schuster & Marion, 1983) and these can be used as guidelines when assessing the immediate prognosis of individual patients. Neither ourselves nor others (Crawford *et al.*, 1988), however, have been able to distinguish any features of the acute illness which influence the likelihood of long term survival; this seems to depend solely on the progress of the underlying malignancy, something which is often difficult to predict before or during intensive care. Despite the high mortality rate, intensive care is therefore justified for patients with acute life-threatening complications of haematological malignancy, unless or until it is clear that there is no prospect of recovery from the acute illness, or that the underlying malignancy cannot be controlled.

Dr E. Yau was in receipt of an Aylwen Bursary through the Special Trustees of St Bartholomew's Hospital.

We would like to thank Ms K. Rowan, UK Apache II study co-ordinator, for helpful advice.

### References

ANGER, B., SCHMEISER, T., SIGEL, H. & HEIMPEL, H. (1987). Intensive care therapy for patients with haematological disease. **30,** 519.

BRUNET, F., LANORE, J.J., DHAINAUT, J.F. & DREYFUS, F. (1990). Is intensive care justified for patients with haematological malignancies? *Intensive Care Med.*, **16,** 291.

CRAWFORD, S.W., SCHWARTZ, D.A., PETERSEN, F. & CLARK, J.C. (1988). Risk factors of mechanical ventilation after marrow transplantation. *Am. Rev. Respir. Dis.*, **137,** 682.

CULLEN, D.J., FERRARA, L.C., BRIGGS, B.A., WALKER, P.F. & GILBERT, J. (1976). Survival hospitalization charges and follow-up results in critically ill patients. *N. Engl. J Med.*, **294,** 982.

DETSKY, A.S., STRICKER, S.C., MULLEY, A.G. & THIBAULT, G.E. (1981). Prognosis, survival, and the expenditure of hospital resources for patients in an intensive care unit. *N. Engl. J. Med.*, **305,** 667.

ESTOPA, R., TORRES-MARTI, A., KASTANOS, N., RIVES, A., AGUSTI-VIDAL, A. & ROZMAN, C. (1984). Acute respiratory failure in severe haematologic disorders. *Crit. Care Med.*, **12,** 26.

HAUSER, M.J., TABAK, J. & BAIER, H. (1982). Survival of patients with cancer in a medical critical care unit. *Arch. Intern. Med.*, **142,** 527.

HUNT, S.M., MCEWEN, J. & MCKENNA, S.P. (1986). *Measuring Health Status.* Croom Helm: London. Pp. 163–202.

JOHNSON, M.H., GORDON, P.W. & FITZGERALD, F.T. (1986). Stratification of prognosis in granulocytopenic patients with haematological malignancies using the APACHE II severity of illness score. *Crit. Care Med.*, **14,** 693.

KNAUS, W.A., DRAPER, E.A., WAGNER, D.P. & ZIMMERMAN, J.E. (1985). APACHE II: a severity of disease classification system. *Crit. Care Med.*, **13,** 818.

LLOYD-THOMAS, A.R., WRIGHT, I., LISTER, T.A. & HINDS, C.J. (1988). Prognosis of patients receiving intensive care for life-threatening medical complications of haematological malignancy. *Br. Med. J.*, **296,** 1025.

PATRICK, D.L., DANIS, N., SOUTHERLAND, L.I. & HONG, G. (1988). Quality of life following intensive care. *J. Gen. Intern. Med.*, **3,** 218.

PETERS, S.G., MEADOWS, J.A. & GRACEY, D.R. (1988). Outcome of respiratory failure in haematologic malignancy. *Chest*, **94**, 99.

SCHUSTER, D.P. & MARION, J.M. (1983). Precedents for a meaningful recovery during treatment in a medical intensive care unit. *Am. J. Med.*, **75**, 402.

SNOW, R.M., MILLER, W.C., RICE, D.L. & ALI, M.K. (1979). Respiratory failure in cancer patients. *JAMA*, **241**, 2039.

TURNBULL, C., CARLON, G., BARON, R., SICHEL, W., YOUNG, C. & HOWLAND, W. (1979). The inverse relationship between cost and survival in the critically ill cancer patient. *Crit. Care Med.*, **7**, 20.

THIBAULT, G.E., MULLEY, A.G., BARNETT, G.O., GOLDSTEIN, R.L., REDER, V.A. & SHERMAN, E.L. (1980). Medical intensive care: indications, interventions, and outcomes. *N. Engl. J. Med.*, **302**, 938.

WANZER, S.H., ADELSTEIN, S.J., CRANFORD, R.E., FEDERMAN, D.D., HOOK, E.D. & MORTEL, C.G. (1984). The physician's responsibility toward hopelessly ill patients. *N. Engl. J. Med.*, **310**, 955.

ZIGMOND, A.S. & SNAITH, R.P. (1983). The Hospital Anxiety and Depression Scale. *Psychiatr. Scand.*, **67**, 361.